# Crystal structure of 6,6,12,12-tetra­chloro­tri­cyclo­[8.2.0.0^4,7^]dodecane-5,11-dione

**DOI:** 10.1107/S2056989015014383

**Published:** 2015-08-06

**Authors:** Esra Turan Akın, Tuncer Hökelek

**Affiliations:** a Department of Chemistry, Atatürk University, 25240, Erzurum, Turkey; b Department of Physics, Hacettepe University, 06800 Beytepe, Ankara, Turkey

**Keywords:** crystal structure, cyclo­octa­diene, fused ring system, hydrogen bonding

## Abstract

The asymmetric unit of the title compound, C_12_H_12_Cl_4_O_2_, contains two crystallographically independent mol­ecules with almost identical conformations (r.m.s. overlay fit for the non-hydrogen atoms = 0.059 Å). In each mol­ecule, the central eight-membered ring has a distorted boat configuration, and two non-planar four-membered rings are fused on either side of the eight-membered ring. A weak C—H⋯O hydrogen bond links the two independent mol­ecules. In the crystal, weak C—H⋯O hydrogen bonds link the mol­ecules into a two-dimensional network parallel to (001).

## Abstract 2

The asymmetric unit contains two independent mol­ecules, each consisting of an eight-membered ring with two four-membered rings fused on either side.

## Chemical context   

The eight-membered-ring cyclic hydro­carbon, 1,5-cyclo­octa­diene (COD), attracts the attention of researchers because of its use as an inter­mediate product in the production of epoxides, suberic acid (1,8-octa­nodioic acid), caprylolactam (8-amino­octa­noic acid lactam) and related chemicals and polymers (Dowd & Zhang, 1991 ▸; Zhang & Dowd, 1992 ▸; Mehta & Rao, 2006 ▸; Brady, 1981 ▸; Ghosez *et al.* 1971 ▸; Brady & Roe, 1971 ▸). COD serves as a useful precursor in the syntheses of other organic compounds and as a ligand in organometallic chemistry (Shriver & Atkins, 1999 ▸).
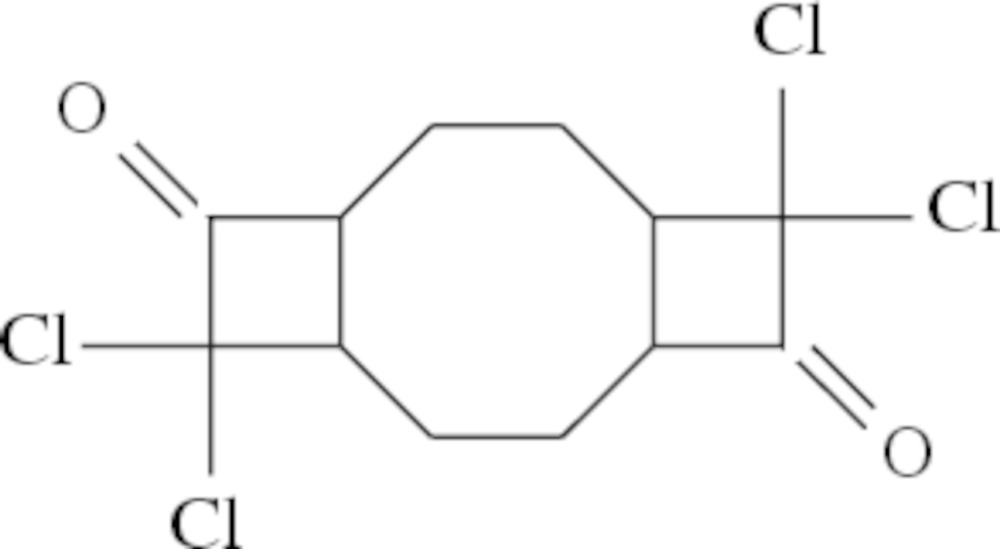


Ketenes, containing *R* and *R*′ groups (where *R*, *R*′ can be hydrogen), and formed cumulene enon systems are reactive compounds. The stability or reactivity of ketenes depends on the electronic structures of the *R* and *R*′ groups. Ketenes providing electron-donating (+I or +M) *R* groups are stable, and their reactivity is low. Electron-attracting ketenes [containing (-I or -M) *R* groups] are less stable and behave in a more unstable manner in reactions.

## Structural commentary   

**Figure 1:**
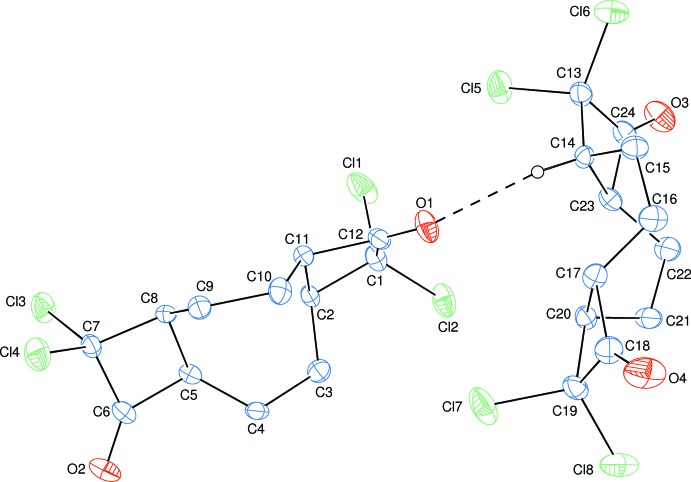
The mol­ecular structure of the title compound with the atom-numbering scheme. Displacement ellipsoids are drawn at the 50% probability level. The inter­molecular C—H ⋯ O hydrogen bond is shown as dashed line. H atoms not involved in hydrogen bonds have been omitted for clarity.

The asymmetric unit of the title compound contains two crystallographically independent mol­ecules (Fig. 1 ▸). Each mol­ecule consists of a central non-planar eight-membered cyclo­octa­diene [*B* (C2–C5/C8–C11) and *E* (C14–C17/C20–C23)] ring system having two non-planar four-membered [*A* (C1/C2/C11/C12), *C* (C5–C8) and *D* (C13/C14/C23/C24), *F* (C17–C20)] rings fused on both sides. A weak C—H⋯O hydrogen bond (Table 1 ▸) links the two independent mol­ecules.

The conformations of the cyclo­octa­diene rings can be clarified from the torsion angles of the rings bonds (Table 2 ▸). The total puckering amplitudes *Q*_T_ of the cyclo­octa­diene rings are 1.632 (3) Å (for ring *B*) and 1.631 (3) Å (for ring *E*). As can also be seen from the distribution of the torsion angles (Table 2 ▸), the asymmetry parameters indicate eight local pseudo twofold axes running along C2⋯C8, C3⋯C9, C4⋯C10, C5⋯C11, the midpoints of C2—C3 and C8—C9, the midpoints of C3—C4 and C9—C10, the midpoints of C4—C5 and C10—C11, the midpoints of C5—C8 and C2—C11 (for ring *B*) and C14⋯C20, C15⋯C21, C16⋯C22, C17⋯C23, the midpoints of C14—C15 and C20—C21, the midpoints of C15—C16 and C21—C22, the midpoints of C16—C17 and C22—C23, the midpoints of C17—C20 and C14—C23 (for ring *E*) (Nardelli, 1983 ▸). In the cyclo­octa­diene rings, the C—C bond distances vary from 1.514 (4) to 1.573 (4) Å (for ring *B*) and 1.508 (4) to 1.573 (4) Å (for ring *E*), while the C—C—C bond angles vary from 114.1 (2) to 121.8 (2)° (for ring *B*) and 114.5 (2) to 121.6 (3)° (for ring *E*). The mean ring C—C bond lengths and C—C—C bond angles are 1.537 (4) Å (for rings *B* and *E*) and 117.0 (4)° (for ring *B*) and 116.9 (3)° (for ring *E*).

In the non-planar four-membered rings (*A*, *C* and *D*, *F*), the (C1/C2/C11) and (C1/C11/C12), (C1/C2/C12) and (C2/C11/C12) (in ring *A*), (C5/C6/C7) and (C5/C7/C8), (C5/C6/C8) and (C6/C7/C8) (in ring *C*), (C13/C14/C23) and (C13/C23/C24), (C13/C14/C24) and (C14/C23/C24) (in ring *D*), (C17/C18/C19) and (C17/C19/C20), (C17/C18/C20) and (C18/C19/C20) (in ring *F*) fragments are oriented at dihedral angles of 155.2 (3), 155.7 (3)° (in ring *A*), 158.4 (3), 158.6 (3)° (in ring *C*), 157.2 (3), 157.5 (3)° (in ring *D*), 155.1 (3), 155.7 (3)° (in ring *F*).

## Supra­molecular features   

**Figure 2:**
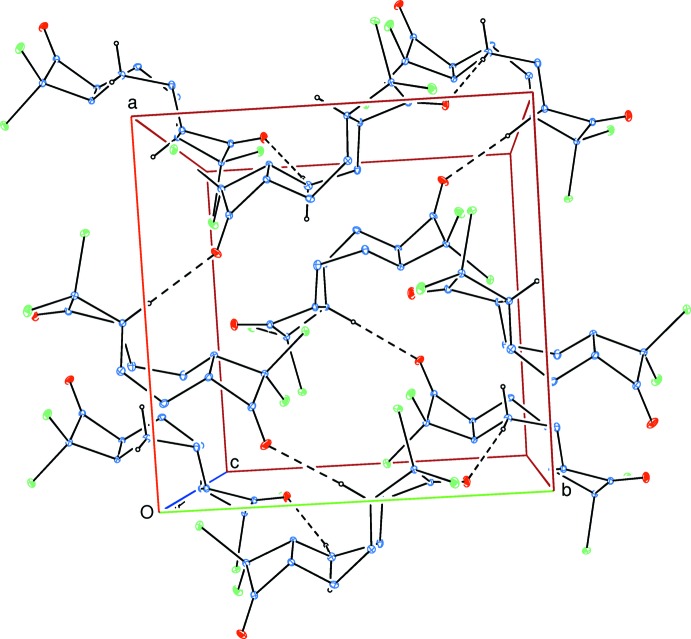
Part of the crystal structure viewed down [001]. Inter­molecular C—H⋯O hydrogen bonds are shown as dashed lines. H atoms not involved in hydrogen bonds have been omitted for clarity.

In the crystal, weak C—H⋯O hydrogen bonds (Table 1 ▸) link the mol­ecules into a two-dimensional network parallel to (001) (Fig. 2 ▸).

## Synthesis and crystallization   

The title compound was synthesized according to a literature method (Bosmajian *et al.* 1964 ▸). For the preparation of the title compound, a mixture of COD (2.00 g, 18.5 mmol) and Zn powder (12.09 g, 184.9 mmol) in absolute ether (15 ml) was stirred for 15 min under a nitro­gen atmosphere. Then, a solution of Cl_3_CCOCl (30.30 g, 64.7 mmol) in absolute ether (20 ml) was added to the mixture over 20 min, and stirred for 20 h under a nitro­gen atmosphere. The reaction mixture was filtered, and the ZnCl_2_ salt was removed. The reaction mixture was extracted with water (3 × 10 ml). The organic phases were combined, and dried over MgSO_4_. The solvent was evaporated and the crude product was eluted in a silica gel (50.00 g) column, and was filtered using ethyl acetate/*n*-hexane (2:8). The obtained solid product (yield; 1.55 g, 25%) was crystallized from CH_2_Cl_2_/*n*-hexane (1:4) solution over two days (m.p. 472–474 K).

## Refinement   

Crystal data, data collection and structure refinement details are summarized in Table 3 ▸. The C-bound H atoms were positioned geometrically with C—H = 0.97 Å (for CH_2_) and 0.98 Å (for CH), and constrained to ride on their parent atoms, *U*_iso_(H) = 1.2*U*_eq_(C).

## Supplementary Material

Crystal structure: contains datablock(s) I, global. DOI: 10.1107/S2056989015014383/xu5862sup1.cif

Structure factors: contains datablock(s) I. DOI: 10.1107/S2056989015014383/xu5862Isup2.hkl

Supporting information file. DOI: 10.1107/S2056989015014383/xu5862Isup3.cml

CCDC reference: 1415865

Additional supporting information:  crystallographic information; 3D view; checkCIF report

## Figures and Tables

**Table 1:** Hydrogen-bond geometry (, )

*D*H*A*	*D*H	H*A*	*D* *A*	*D*H*A*
C3H3*B*O2^i^	0.97	2.57	3.473(4)	154
C8H8O4^ii^	0.98	2.43	3.406(4)	176
C14H14O1	0.98	2.38	3.342(4)	168

Symmetry codes: (i) 
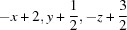
; (ii) 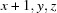.

**Table 2:** Selected torsion angles ()

C11C2C3C4	67.5(3)	C23C14C15C16	65.6(4)
C2C3C4C5	24.0(4)	C15C14C23C22	21.1(4)
C8C5C4C3	77.3(3)	C17C16C15C14	25.8(4)
C9C8C5C4	19.1(4)	C20C17C16C15	76.5(4)
C5C8C9C10	65.3(3)	C21C20C17C16	21.6(4)
C8C9C10C11	24.6(4)	C17C20C21C22	67.5(4)
C10C11C2C3	22.1(4)	C20C21C22C23	23.7(4)
C2C11C10C9	75.4(3)	C14C23C22C21	75.6(4)

**Table 3:** Experimental details

Crystal data	
Chemical formula	C_12_H_12_Cl_4_O_2_
*M*_r_	330.02
Crystal system, space group	Monoclinic, *P*2_1_/*c*
Temperature (K)	296
*a*, *b*, *c* ()	10.9786(3), 10.9374(3), 23.5429(5)
()	97.554(2)
*V* (^3^)	2802.43(12)
*Z*	8
Radiation type	Mo *K*
(mm^1^)	0.83
Crystal size (mm)	0.11 0.10 0.07

Data collection	
Diffractometer	Bruker Kappa APEXII CCD area-detector
Absorption correction	Multi-scan (*SADABS*; Bruker, 2012 ▸)
*T*_min_, *T*_max_	0.901, 0.933
No. of measured, independent and observed [*I* > 2(*I*)] reflections	64865, 6994, 4542
*R*_int_	0.069
(sin /)_max_ (^1^)	0.668

Refinement	
*R*[*F* ^2^ > 2(*F* ^2^)], *wR*(*F* ^2^), *S*	0.064, 0.131, 1.10
No. of reflections	6994
No. of parameters	325
H-atom treatment	H-atom parameters constrained
_max_, _min_ (e ^3^)	0.70, 0.54

Computer programs: *APEX2* and *SAINT* (Bruker, 2012 ▸), *SHELXS97* and *SHELXL97* (Sheldrick, 2008 ▸), *ORTEP-3 for Windows* and *WinGX* (Farrugia, 2012 ▸) and *PLATON* (Spek, 2009 ▸).

## supplementary crystallographic information

### Crystal data

**Table:**

C_12_H_12_Cl_4_O_2_	*F*(000) = 1344
*M**_r_* = 330.02	*D*_x_ = 1.564 Mg m^−^^3^
Monoclinic, *P*2_1_/*c*	Mo *K*α radiation, λ = 0.71073 Å
Hall symbol: -P 2ybc	Cell parameters from 9888 reflections
*a* = 10.9786 (3) Å	θ = 3.2–27.5°
*b* = 10.9374 (3) Å	µ = 0.83 mm^−^^1^
*c* = 23.5429 (5) Å	*T* = 296 K
β = 97.554 (2)°	Block, colorless
*V* = 2802.43 (12) Å^3^	0.11 × 0.10 × 0.07 mm
*Z* = 8	

### Data collection

**Table:**

Bruker Kappa APEXII CCD area-detector diffractometer	6994 independent reflections
Radiation source: fine-focus sealed tube	4542 reflections with *I* > 2σ(*I*)
Graphite monochromator	*R*_int_ = 0.069
φ and ω scans	θ_max_ = 28.4°, θ_min_ = 3.0°
Absorption correction: multi-scan (*SADABS*; Bruker, 2012)	*h* = −14→13
*T*_min_ = 0.901, *T*_max_ = 0.933	*k* = −14→14
64865 measured reflections	*l* = −31→31

### Refinement

**Table:**

Refinement on *F*^2^	Primary atom site location: structure-invariant direct methods
Least-squares matrix: full	Secondary atom site location: difference Fourier map
*R*[*F*^2^ > 2σ(*F*^2^)] = 0.064	Hydrogen site location: inferred from neighbouring sites
*wR*(*F*^2^) = 0.131	H-atom parameters constrained
*S* = 1.10	*w* = 1/[σ^2^(*F*_o_^2^) + (0.0326*P*)^2^ + 3.8912*P*] where *P* = (*F*_o_^2^ + 2*F*_c_^2^)/3
6994 reflections	(Δ/σ)_max_ = 0.001
325 parameters	Δρ_max_ = 0.70 e Å^−^^3^
0 restraints	Δρ_min_ = −0.54 e Å^−^^3^

### Special details

**Table:**

Geometry. All esds (except the esd in the dihedral angle between two l.s. planes) are estimated using the full covariance matrix. The cell esds are taken into account individually in the estimation of esds in distances, angles and torsion angles; correlations between esds in cell parameters are only used when they are defined by crystal symmetry. An approximate (isotropic) treatment of cell esds is used for estimating esds involving l.s. planes.
Refinement. Refinement of F^2^ against ALL reflections. The weighted R-factor wR and goodness of fit S are based on F^2^, conventional R-factors R are based on F, with F set to zero for negative F^2^. The threshold expression of F^2^ > 2sigma(F^2^) is used only for calculating R-factors(gt) etc. and is not relevant to the choice of reflections for refinement. R-factors based on F^2^ are statistically about twice as large as those based on F, and R- factors based on ALL data will be even larger.

### Fractional atomic coordinates and isotropic or equivalent isotropic displacement parameters (Å^2^)

**Table:**

	*x*	*y*	*z*	*U*_iso_*/*U*_eq_	
Cl1	0.93872 (9)	0.43272 (9)	0.66192 (5)	0.0694 (3)	
Cl2	0.74549 (9)	0.35318 (9)	0.72561 (4)	0.0574 (3)	
Cl3	1.22527 (7)	−0.11648 (9)	0.60649 (4)	0.0539 (2)	
Cl4	1.03076 (9)	−0.25182 (8)	0.53791 (4)	0.0525 (2)	
Cl5	0.72656 (8)	0.66388 (9)	0.59660 (6)	0.0717 (3)	
Cl6	0.53149 (10)	0.80518 (9)	0.53202 (4)	0.0598 (3)	
Cl7	0.44151 (9)	0.11622 (9)	0.64870 (6)	0.0770 (4)	
Cl8	0.25781 (10)	0.19145 (10)	0.71898 (4)	0.0653 (3)	
O1	0.6556 (2)	0.3265 (2)	0.58919 (10)	0.0511 (6)	
O2	1.0164 (2)	−0.2842 (2)	0.67506 (11)	0.0537 (6)	
O3	0.5260 (3)	0.8322 (2)	0.66995 (11)	0.0600 (7)	
O4	0.1521 (2)	0.2303 (3)	0.58563 (12)	0.0677 (8)	
C1	0.8295 (3)	0.3158 (3)	0.66906 (14)	0.0396 (7)	
C2	0.8854 (3)	0.1854 (3)	0.66500 (12)	0.0299 (6)	
H2	0.9753	0.1858	0.6726	0.036*	
C3	0.8287 (3)	0.0903 (3)	0.70038 (13)	0.0370 (7)	
H3A	0.7401	0.0991	0.6936	0.044*	
H3B	0.8547	0.1071	0.7406	0.044*	
C4	0.8614 (3)	−0.0435 (3)	0.68815 (13)	0.0351 (7)	
H4A	0.8672	−0.0894	0.7237	0.042*	
H4B	0.7951	−0.0786	0.6619	0.042*	
C5	0.9808 (3)	−0.0583 (3)	0.66279 (12)	0.0299 (6)	
H5	1.0465	−0.0141	0.6865	0.036*	
C6	1.0233 (3)	−0.1884 (3)	0.65266 (13)	0.0334 (7)	
C7	1.0648 (3)	−0.1495 (3)	0.59541 (13)	0.0326 (7)	
C8	0.9881 (2)	−0.0305 (3)	0.59796 (11)	0.0268 (6)	
H8	1.0375	0.0425	0.5933	0.032*	
C9	0.8675 (3)	−0.0282 (3)	0.55768 (12)	0.0319 (6)	
H9A	0.8264	−0.1061	0.5605	0.038*	
H9B	0.8861	−0.0204	0.5187	0.038*	
C10	0.7780 (3)	0.0745 (3)	0.56886 (13)	0.0359 (7)	
H10A	0.7336	0.1001	0.5324	0.043*	
H10B	0.7184	0.0417	0.5918	0.043*	
C11	0.8375 (3)	0.1859 (3)	0.59901 (12)	0.0309 (6)	
H11	0.9022	0.2166	0.5778	0.037*	
C12	0.7531 (3)	0.2904 (3)	0.61064 (13)	0.0345 (7)	
C13	0.5667 (3)	0.6999 (3)	0.58827 (14)	0.0394 (7)	
C14	0.4886 (2)	0.5814 (3)	0.59130 (12)	0.0304 (6)	
H14	0.5354	0.5079	0.5843	0.036*	
C15	0.3647 (3)	0.5847 (3)	0.55390 (13)	0.0384 (7)	
H15A	0.3783	0.5788	0.5141	0.046*	
H15B	0.3265	0.6632	0.5590	0.046*	
C16	0.2746 (3)	0.4827 (3)	0.56630 (14)	0.0408 (8)	
H16A	0.2204	0.5146	0.5921	0.049*	
H16B	0.2243	0.4610	0.5307	0.049*	
C17	0.3358 (3)	0.3679 (3)	0.59241 (13)	0.0343 (7)	
H17	0.3964	0.3386	0.5685	0.041*	
C18	0.2521 (3)	0.2633 (3)	0.60474 (15)	0.0420 (8)	
C19	0.3348 (3)	0.2323 (3)	0.66063 (15)	0.0420 (8)	
C20	0.3916 (3)	0.3620 (3)	0.65730 (13)	0.0337 (7)	
H20	0.4816	0.3600	0.6624	0.040*	
C21	0.3421 (3)	0.4551 (3)	0.69642 (13)	0.0408 (8)	
H21A	0.3726	0.4344	0.7358	0.049*	
H21B	0.2532	0.4481	0.6920	0.049*	
C22	0.3756 (3)	0.5896 (3)	0.68587 (14)	0.0430 (8)	
H22A	0.3070	0.6279	0.6623	0.052*	
H22B	0.3872	0.6320	0.7224	0.052*	
C23	0.4896 (3)	0.6061 (3)	0.65719 (12)	0.0331 (7)	
H23	0.5577	0.5610	0.6787	0.040*	
C24	0.5308 (3)	0.7358 (3)	0.64670 (14)	0.0397 (8)	

### Atomic displacement parameters (Å^2^)

**Table:**

	*U*^11^	*U*^22^	*U*^33^	*U*^12^	*U*^13^	*U*^23^
Cl1	0.0513 (5)	0.0330 (5)	0.1188 (9)	−0.0081 (4)	−0.0081 (5)	0.0036 (5)
Cl2	0.0661 (6)	0.0528 (6)	0.0511 (5)	0.0209 (5)	−0.0012 (4)	−0.0169 (4)
Cl3	0.0289 (4)	0.0472 (5)	0.0861 (7)	0.0055 (4)	0.0089 (4)	−0.0021 (5)
Cl4	0.0624 (6)	0.0450 (5)	0.0501 (5)	0.0064 (4)	0.0079 (4)	−0.0147 (4)
Cl5	0.0334 (5)	0.0460 (6)	0.1380 (10)	−0.0049 (4)	0.0196 (5)	0.0034 (6)
Cl6	0.0738 (6)	0.0465 (6)	0.0594 (6)	−0.0112 (5)	0.0106 (5)	0.0138 (4)
Cl7	0.0478 (5)	0.0304 (5)	0.1492 (11)	0.0037 (4)	−0.0001 (6)	−0.0076 (6)
Cl8	0.0705 (6)	0.0599 (7)	0.0636 (6)	−0.0214 (5)	0.0013 (5)	0.0226 (5)
O1	0.0460 (14)	0.0513 (16)	0.0524 (14)	0.0212 (12)	−0.0069 (11)	0.0002 (12)
O2	0.0708 (17)	0.0295 (14)	0.0590 (15)	−0.0002 (12)	0.0019 (13)	0.0135 (12)
O3	0.0795 (19)	0.0327 (15)	0.0642 (16)	−0.0003 (13)	−0.0036 (14)	−0.0151 (13)
O4	0.0529 (16)	0.0673 (19)	0.0757 (18)	−0.0307 (14)	−0.0185 (13)	0.0128 (15)
C1	0.0358 (17)	0.0296 (18)	0.0515 (19)	0.0038 (13)	−0.0014 (14)	−0.0037 (15)
C2	0.0259 (14)	0.0229 (15)	0.0402 (16)	0.0026 (11)	0.0007 (12)	−0.0015 (12)
C3	0.0425 (17)	0.041 (2)	0.0286 (15)	0.0015 (14)	0.0076 (13)	−0.0013 (14)
C4	0.0475 (18)	0.0252 (17)	0.0348 (16)	−0.0022 (13)	0.0140 (13)	0.0056 (13)
C5	0.0351 (15)	0.0250 (15)	0.0283 (15)	−0.0009 (12)	−0.0005 (12)	0.0021 (12)
C6	0.0322 (15)	0.0247 (17)	0.0406 (17)	0.0004 (12)	−0.0048 (13)	0.0026 (13)
C7	0.0311 (15)	0.0248 (16)	0.0423 (17)	0.0025 (12)	0.0059 (12)	−0.0032 (13)
C8	0.0271 (14)	0.0224 (15)	0.0317 (15)	−0.0018 (11)	0.0066 (11)	0.0038 (12)
C9	0.0345 (15)	0.0339 (17)	0.0270 (15)	0.0018 (13)	0.0028 (12)	−0.0005 (13)
C10	0.0289 (15)	0.044 (2)	0.0331 (16)	0.0055 (13)	−0.0025 (12)	−0.0001 (14)
C11	0.0290 (15)	0.0288 (16)	0.0357 (16)	0.0040 (12)	0.0067 (12)	0.0055 (13)
C12	0.0353 (16)	0.0272 (17)	0.0406 (17)	0.0023 (13)	0.0039 (13)	0.0070 (13)
C13	0.0330 (16)	0.0301 (18)	0.056 (2)	−0.0012 (13)	0.0068 (14)	0.0015 (15)
C14	0.0292 (14)	0.0255 (16)	0.0378 (16)	−0.0004 (12)	0.0095 (12)	−0.0051 (13)
C15	0.0413 (17)	0.0403 (19)	0.0329 (16)	−0.0035 (14)	0.0017 (13)	0.0029 (14)
C16	0.0344 (17)	0.046 (2)	0.0399 (18)	−0.0084 (14)	−0.0054 (13)	0.0028 (15)
C17	0.0318 (15)	0.0326 (17)	0.0385 (17)	−0.0062 (13)	0.0047 (12)	−0.0078 (13)
C18	0.0371 (18)	0.0345 (19)	0.054 (2)	−0.0105 (14)	0.0034 (15)	−0.0074 (15)
C19	0.0318 (16)	0.0288 (18)	0.064 (2)	−0.0032 (13)	−0.0002 (15)	0.0069 (16)
C20	0.0275 (14)	0.0285 (17)	0.0441 (17)	−0.0005 (12)	0.0016 (12)	−0.0006 (13)
C21	0.052 (2)	0.0332 (19)	0.0382 (18)	−0.0039 (15)	0.0111 (15)	0.0043 (14)
C22	0.061 (2)	0.037 (2)	0.0333 (17)	0.0035 (16)	0.0168 (15)	−0.0049 (14)
C23	0.0384 (16)	0.0235 (16)	0.0352 (16)	−0.0007 (13)	−0.0032 (13)	−0.0040 (13)
C24	0.0386 (17)	0.0307 (19)	0.0466 (19)	0.0007 (14)	−0.0064 (14)	−0.0030 (15)

### Geometric parameters (Å, º)

**Table:**

Cl1—C1	1.776 (3)	C11—H11	0.9800
Cl2—C1	1.764 (3)	C12—O1	1.189 (3)
Cl3—C7	1.783 (3)	C12—C1	1.539 (4)
Cl4—C7	1.758 (3)	C12—C11	1.519 (4)
Cl5—C13	1.784 (3)	C14—C13	1.561 (4)
Cl6—C13	1.759 (3)	C14—C15	1.521 (4)
Cl7—C19	1.775 (3)	C14—C23	1.573 (4)
Cl8—C19	1.763 (4)	C14—H14	0.9800
C2—C1	1.561 (4)	C15—H15A	0.9700
C2—C3	1.516 (4)	C15—H15B	0.9700
C2—H2	0.9800	C16—C15	1.544 (4)
C3—C4	1.542 (4)	C16—H16A	0.9700
C3—H3A	0.9700	C16—H16B	0.9700
C3—H3B	0.9700	C17—C16	1.515 (4)
C4—H4A	0.9700	C17—C18	1.519 (4)
C4—H4B	0.9700	C17—H17	0.9800
C5—C4	1.519 (4)	C18—O4	1.187 (4)
C5—H5	0.9800	C18—C19	1.535 (5)
C6—O2	1.180 (4)	C20—C17	1.571 (4)
C6—C5	1.526 (4)	C20—C19	1.556 (4)
C6—C7	1.538 (4)	C20—C21	1.520 (4)
C8—C5	1.569 (4)	C20—H20	0.9800
C8—C7	1.557 (4)	C21—C22	1.544 (5)
C8—C9	1.524 (4)	C21—H21A	0.9700
C8—H8	0.9800	C21—H21B	0.9700
C9—C10	1.538 (4)	C22—H22A	0.9700
C9—H9A	0.9700	C22—H22B	0.9700
C9—H9B	0.9700	C23—C22	1.508 (4)
C10—H10A	0.9700	C23—C24	1.520 (4)
C10—H10B	0.9700	C23—H23	0.9800
C11—C2	1.573 (4)	C24—O3	1.192 (4)
C11—C10	1.514 (4)	C24—C13	1.531 (5)
			
Cl2—C1—Cl1	109.33 (18)	Cl6—C13—Cl5	110.07 (17)
C2—C1—Cl1	112.2 (2)	C14—C13—Cl5	110.5 (2)
C2—C1—Cl2	120.4 (2)	C14—C13—Cl6	120.7 (2)
C12—C1—Cl1	109.9 (2)	C24—C13—Cl5	108.9 (2)
C12—C1—Cl2	116.0 (2)	C24—C13—Cl6	116.8 (2)
C12—C1—C2	87.3 (2)	C24—C13—C14	87.9 (2)
C1—C2—C11	88.5 (2)	C13—C14—C23	88.3 (2)
C1—C2—H2	112.2	C13—C14—H14	111.8
C3—C2—C1	113.6 (2)	C15—C14—C13	114.2 (3)
C3—C2—C11	115.9 (2)	C15—C14—C23	117.1 (2)
C3—C2—H2	112.2	C15—C14—H14	111.8
C11—C2—H2	112.2	C23—C14—H14	111.8
C2—C3—C4	115.2 (2)	C14—C15—C16	114.8 (3)
C2—C3—H3A	108.5	C14—C15—H15A	108.6
C2—C3—H3B	108.5	C14—C15—H15B	108.6
C4—C3—H3A	108.5	C16—C15—H15A	108.6
C4—C3—H3B	108.5	C16—C15—H15B	108.6
H3A—C3—H3B	107.5	H15A—C15—H15B	107.5
C3—C4—H4A	108.7	C15—C16—H16A	108.6
C3—C4—H4B	108.7	C15—C16—H16B	108.6
C5—C4—C3	114.1 (2)	C17—C16—C15	114.5 (2)
C5—C4—H4A	108.7	C17—C16—H16A	108.6
C5—C4—H4B	108.7	C17—C16—H16B	108.6
H4A—C4—H4B	107.6	H16A—C16—H16B	107.6
C4—C5—C6	117.3 (2)	C16—C17—C18	117.0 (3)
C4—C5—C8	121.3 (2)	C16—C17—C20	121.6 (3)
C4—C5—H5	109.4	C16—C17—H17	109.7
C6—C5—C8	88.5 (2)	C18—C17—C20	87.2 (2)
C6—C5—H5	109.4	C18—C17—H17	109.7
C8—C5—H5	109.4	C20—C17—H17	109.7
O2—C6—C5	136.0 (3)	O4—C18—C17	135.2 (3)
O2—C6—C7	132.9 (3)	O4—C18—C19	132.2 (3)
C5—C6—C7	90.7 (2)	C17—C18—C19	91.5 (2)
Cl4—C7—Cl3	110.34 (16)	Cl8—C19—Cl7	109.62 (18)
C6—C7—Cl3	109.2 (2)	C18—C19—Cl7	110.5 (2)
C6—C7—Cl4	116.2 (2)	C18—C19—Cl8	115.7 (2)
C6—C7—C8	88.4 (2)	C18—C19—C20	87.2 (2)
C8—C7—Cl3	110.7 (2)	C20—C19—Cl7	111.5 (2)
C8—C7—Cl4	120.0 (2)	C20—C19—Cl8	120.5 (2)
C5—C8—H8	111.5	C17—C20—H20	112.2
C7—C8—C5	88.4 (2)	C19—C20—C17	88.8 (2)
C7—C8—H8	111.5	C19—C20—H20	112.2
C9—C8—C5	117.2 (2)	C21—C20—C17	115.9 (3)
C9—C8—C7	114.8 (2)	C21—C20—C19	113.7 (3)
C9—C8—H8	111.5	C21—C20—H20	112.2
C8—C9—C10	115.3 (2)	C20—C21—C22	115.4 (3)
C8—C9—H9A	108.4	C20—C21—H21A	108.4
C8—C9—H9B	108.4	C22—C21—H21A	108.4
C10—C9—H9A	108.4	C20—C21—H21B	108.4
C10—C9—H9B	108.4	C22—C21—H21B	108.4
H9A—C9—H9B	107.5	H21A—C21—H21B	107.5
C9—C10—H10A	108.5	C21—C22—H22A	108.6
C9—C10—H10B	108.5	C21—C22—H22B	108.6
C11—C10—C9	115.0 (2)	C23—C22—C21	114.5 (3)
C11—C10—H10A	108.5	C23—C22—H22A	108.6
C11—C10—H10B	108.5	C23—C22—H22B	108.6
H10A—C10—H10B	107.5	H22A—C22—H22B	107.6
C2—C11—H11	109.5	C14—C23—H23	109.3
C10—C11—C2	121.8 (2)	C22—C23—H23	109.3
C10—C11—C12	117.0 (2)	C22—C23—C14	121.5 (2)
C10—C11—H11	109.5	C22—C23—C24	117.8 (3)
C12—C11—C2	87.5 (2)	C24—C23—C14	87.9 (2)
C12—C11—H11	109.5	C24—C23—H23	109.3
O1—C12—C1	132.4 (3)	O3—C24—C13	132.2 (3)
O1—C12—C11	135.2 (3)	O3—C24—C23	136.0 (3)
C11—C12—C1	91.3 (2)	C23—C24—C13	91.4 (2)
			
C3—C2—C1—Cl1	148.8 (2)	C15—C14—C13—Cl5	−147.1 (2)
C3—C2—C1—Cl2	18.0 (3)	C15—C14—C13—Cl6	−16.8 (4)
C3—C2—C1—C12	−100.8 (3)	C15—C14—C13—C24	103.5 (3)
C11—C2—C1—Cl1	−93.4 (2)	C23—C14—C13—Cl5	93.7 (2)
C11—C2—C1—Cl2	135.7 (2)	C23—C14—C13—Cl6	−135.9 (2)
C11—C2—C1—C12	17.0 (2)	C23—C14—C13—C24	−15.7 (2)
C1—C2—C3—C4	168.0 (3)	C13—C14—C15—C16	−166.8 (3)
C11—C2—C3—C4	67.5 (3)	C23—C14—C15—C16	−65.6 (4)
C2—C3—C4—C5	24.0 (4)	C13—C14—C23—C22	137.5 (3)
C6—C5—C4—C3	176.6 (3)	C13—C14—C23—C24	15.8 (2)
C8—C5—C4—C3	−77.3 (3)	C15—C14—C23—C22	21.1 (4)
O2—C6—C5—C4	−32.6 (5)	C15—C14—C23—C24	−100.6 (3)
O2—C6—C5—C8	−157.4 (4)	C17—C16—C15—C14	−25.8 (4)
C7—C6—C5—C4	140.0 (3)	C18—C17—C16—C15	−179.0 (3)
C7—C6—C5—C8	15.2 (2)	C20—C17—C16—C15	76.5 (4)
O2—C6—C7—Cl3	−90.9 (4)	C16—C17—C18—O4	26.3 (6)
O2—C6—C7—C8	157.7 (4)	C16—C17—C18—C19	−141.8 (3)
O2—C6—C7—Cl4	34.7 (4)	C20—C17—C18—O4	150.7 (4)
C5—C6—C7—Cl3	96.1 (2)	C20—C17—C18—C19	−17.4 (2)
C5—C6—C7—Cl4	−138.3 (2)	O4—C18—C19—Cl7	97.0 (4)
C5—C6—C7—C8	−15.3 (2)	O4—C18—C19—Cl8	−28.4 (5)
C7—C8—C5—C4	−136.4 (3)	O4—C18—C19—C20	−151.1 (4)
C7—C8—C5—C6	−15.0 (2)	C17—C18—C19—Cl7	−94.4 (2)
C9—C8—C5—C4	−19.1 (4)	C17—C18—C19—Cl8	140.3 (2)
C9—C8—C5—C6	102.2 (3)	C17—C18—C19—C20	17.6 (2)
C5—C8—C7—Cl3	−95.1 (2)	C19—C20—C17—C16	137.5 (3)
C5—C8—C7—Cl4	134.5 (2)	C19—C20—C17—C18	17.2 (2)
C5—C8—C7—C6	14.9 (2)	C21—C20—C17—C16	21.6 (4)
C9—C8—C7—Cl3	145.5 (2)	C21—C20—C17—C18	−98.7 (3)
C9—C8—C7—Cl4	15.1 (3)	C17—C20—C19—Cl7	94.0 (2)
C9—C8—C7—C6	−104.5 (2)	C17—C20—C19—Cl8	−135.4 (2)
C7—C8—C9—C10	167.1 (2)	C17—C20—C19—C18	−17.0 (2)
C5—C8—C9—C10	65.3 (3)	C21—C20—C19—Cl7	−148.1 (2)
C8—C9—C10—C11	24.6 (4)	C21—C20—C19—Cl8	−17.5 (4)
C10—C11—C2—C1	−137.8 (3)	C21—C20—C19—C18	100.9 (3)
C10—C11—C2—C3	−22.1 (4)	C17—C20—C21—C22	−67.5 (4)
C12—C11—C2—C1	−17.2 (2)	C19—C20—C21—C22	−168.3 (3)
C12—C11—C2—C3	98.5 (3)	C20—C21—C22—C23	−23.7 (4)
C2—C11—C10—C9	−75.4 (3)	C14—C23—C22—C21	75.6 (4)
C12—C11—C10—C9	179.5 (2)	C24—C23—C22—C21	−178.4 (3)
O1—C12—C1—Cl1	−96.0 (4)	C14—C23—C24—O3	156.3 (4)
O1—C12—C1—Cl2	28.6 (5)	C14—C23—C24—C13	−16.1 (2)
O1—C12—C1—C2	151.4 (4)	C22—C23—C24—O3	31.4 (5)
C11—C12—C1—Cl1	95.0 (2)	C22—C23—C24—C13	−141.0 (3)
C11—C12—C1—Cl2	−140.4 (2)	O3—C24—C13—Cl5	92.4 (4)
C11—C12—C1—C2	−17.6 (2)	O3—C24—C13—Cl6	−33.0 (5)
O1—C12—C11—C2	−151.0 (4)	O3—C24—C13—C14	−156.6 (4)
O1—C12—C11—C10	−26.2 (5)	C23—C24—C13—Cl5	−94.7 (2)
C1—C12—C11—C2	17.5 (2)	C23—C24—C13—Cl6	139.9 (2)
C1—C12—C11—C10	142.3 (3)	C23—C24—C13—C14	16.2 (2)

### Hydrogen-bond geometry (Å, º)

**Table:**

*D*—H···*A*	*D*—H	H···*A*	*D*···*A*	*D*—H···*A*
C3—H3*B*···O2^i^	0.97	2.57	3.473 (4)	154
C8—H8···O4^ii^	0.98	2.43	3.406 (4)	176
C14—H14···O1	0.98	2.38	3.342 (4)	168

Symmetry codes: (i) −*x*+2, *y*+1/2, −*z*+3/2; (ii) *x*+1, *y*, *z*.
